# Multi-modal retinal scanning to measure retinal thickness and peripheral blood vessels in multiple sclerosis

**DOI:** 10.1038/s41598-022-24312-4

**Published:** 2022-11-28

**Authors:** Thomas Pearson, Yingdi Chen, Baljean Dhillon, Siddharthan Chandran, Jano van Hemert, Tom MacGillivray

**Affiliations:** 1 Centre for Clinical Brain Sciences, University of Edinburgh, Edinburgh, UK; 2 Anne Rowling Regenerative Neurology Clinic, University of Edinburgh, Edinburgh, UK; 3 Princess Alexandra Eye Pavilion, NHS Lothian, Edinburgh, UK; 4 Optos Plc, Dunfermline, KY11 8GR UK

**Keywords:** Optical imaging, Blood-brain barrier, Multiple sclerosis, Diagnostic markers, Predictive markers, Multiple sclerosis

## Abstract

Our purpose was to investigate changes to the retina in multiple sclerosis (MS) using established and novel modes of retinal image acquisition and analysis. 72 participants with MS and 80 healthy volunteers underwent retinal scanning with optical coherence tomography (OCT) and ultra-widefield (UWF) scanning laser ophthalmoscopy (SLO), over a two-year period. Changes in retinal nerve fibre layer (RNFL) thickness, macular volume and retinal blood vessel diameter were measured and parameters were then tested for associations with MS. Measurements from OCT showed that individuals with MS had a thinner RNFL and reduced macular volume when compared to healthy volunteers. On UWF images, participants with MS had reduced arterial widths in the inferior nasal quadrant of both eyes and reduced venous widths in the inferior nasal quadrant of right eyes. Longitudinal analysis showed that participants with MS had an accelerated annual rate of RNFL thinning in several regions of the retina. In conclusion, the assessment of OCT showed thinning of the RNFL and macula in concordance with previous reports on MS, while analysis of blood vessels in the retinal periphery from UWF-SLO images revealed novel changes.

## Introduction

Multiple Sclerosis (MS) is a chronic neurodegenerative disease; with over 2.8 million sufferers worldwide, it is the most common cause of non-traumatic neurological disability in young adults^1,2^. The disease is characterised by the demyelination (destruction of the insulating fat surrounding a nerve) and eventual degeneration of axons within the central nervous system (CNS). One of the earliest pathological manifestations of MS is an increased permeability of the blood vessels that supply the CNS (the blood–brain barrier), which subsequently allows immune cells to cause inflammation within the CNS^3–6^. The question as to how neurodegeneration occurs in isolation has yet to be definitively answered in the context of MS.

A diagnosis of MS increasingly relies on a range of diagnostic procedures (e.g., MRI and cerebrospinal fluid analysis) but a clinical picture consistent with MS is essential in recognising early signs of disease and making a definitive diagnosis^7,8^. The variable pattern of disease between individuals can result in delays to diagnosis and uncertain prognoses, potentially preventing patients from receiving the benefit of disease-modifying treatments early in their disease course when these are most effective^9,10^. The eye offers a potential solution as a non-invasive method of capturing images of the CNS and retinal vasculature at high resolution via the anterior visual pathways, where detecting changes early could increase understanding of the initial mechanisms of disease (i.e., inflammation and neurodegeneration), thus aiding the creation of new targeted treatments and therapies^11,12^.

Optical coherence tomography (OCT) quantifies changes to macular volume and retinal nerve fibre layer (RNFL) thickness that may act as surrogates for similar changes in the brain^9,13–15^. A meta-analysis of 3100 eyes found a mean RNFL thickness reduction of 7 μm in MS patients who had never reported a clinical episode of optic neuritis (an acute inflammation of the optic nerve with an identical pathophysiology to that of MS) when compared to healthy controls, with a large multi-centre study seeing similar reductions^16^. Although data has suggested that thinning of the RNFL and macular volume occurs gradually as the disease progresses, regression analysis in one study indicated that the thinning occurred at a greater rate early in the disease^17,18^. Changes in RNFL thickness are greatest in the temporal retina, to the extent that studies have differentiated primary progressive MS patients from those with secondary progressive disease (a progressive course following an initial relapsing–remitting presentation) using temporal RNFL thickness reductions alone^19^. Macular volume has also been shown to decrease in patients with MS^20^.

Studies that feature analysis of the retinal vasculature are scarce in comparison to those that use OCT. This is despite retinal changes not being exclusive to the CNS with, for example, individuals with a higher Expanded Disability Status Scale (EDSS) score more likely to suffer from retinal periphlebitis (an inflammation of the peripheral retinal vasculature), supporting its use as a biomarker of disease progression^21,22^. To our best knowledge, this study represents the first investigation of blood vessels in the peripheral retina alongside established measures of RNFL thickness and macular volume in patients with MS, providing information on the interaction between inflammatory and neurodegenerative disease mechanisms.

## Materials and methods

Participants aged 18 years or older were recruited from the Anne Rowling Regenerative Neurology Clinic (University of Edinburgh) following written informed consent, having all previously received a formal diagnosis of MS- confirmed by the treating consultant neurologist as fulfilling the most recent McDonald Criteria at the time of diagnosis. Healthy volunteers were recruited locally by written invitation, with the spouses of MS patients (who would regularly attend clinics with their partner) also invited to participate in the study. Ethical approval was granted from the NRES Committee London Southeast, which followed the tenants of the Declaration of Helsinki to assess the study’s protocol. Exclusion criteria included any individuals who had undergone retinal surgery, had severe myopia (in line with technical specifications for the imaging devices used, we excluded participants with a refractive error of − 10 dioptres or more), retinal pathologies, or any pathologies that would compromise the ability to make accurate retinal measurements in either eye e.g., glaucoma. Due to the low sample size of participants with a clinical history of optic neuritis (OD: N = 9, OS: N = 10), only MS participants without a clinical history of optic neuritis were included in the analysis.

OCT imaging was conducted using a Heidelberg SPECTRALIS (Heidelberg Engineering, Heidelberg, Germany) to acquire scans of the posterior pole (30° × 25° high speed mode comprising 61 vertical B-scans centred on the fovea) to measure macular volume, and the peripapillary region to measure RNFL thickness (at a radius of 3.4 mm from the optic disc’s centre) in the nine Early Treatment Diabetic Retinopathy Study (ETDRS) sectors^23^. Automatic real time (ART- a measure of how many individual scans were averaged to create a single composite image) was set at 100 for peripapillary scans and 12 for posterior pole scans. A minimum quality score (a measure of signal strength given by the manufacturer’s proprietary software) of 25 was used to ensure adequate segmentation of the anatomical layers of the retina. RNFL and macular volume segmentations were produced by the device’s own software (HEYEX, software version 6.5, Heidelberg Engineering, Heidelberg, Germany) with manual correction by a single operator (author TP) where needed. Images were selected through careful assessment of quality score, ART score and quality of segmentation in conjunction with OSCAR-IB criteria^24^. Each participant’s baseline scan was referenced anatomically to allow for direct comparison with future follow-up scans.

**Figure 1:**
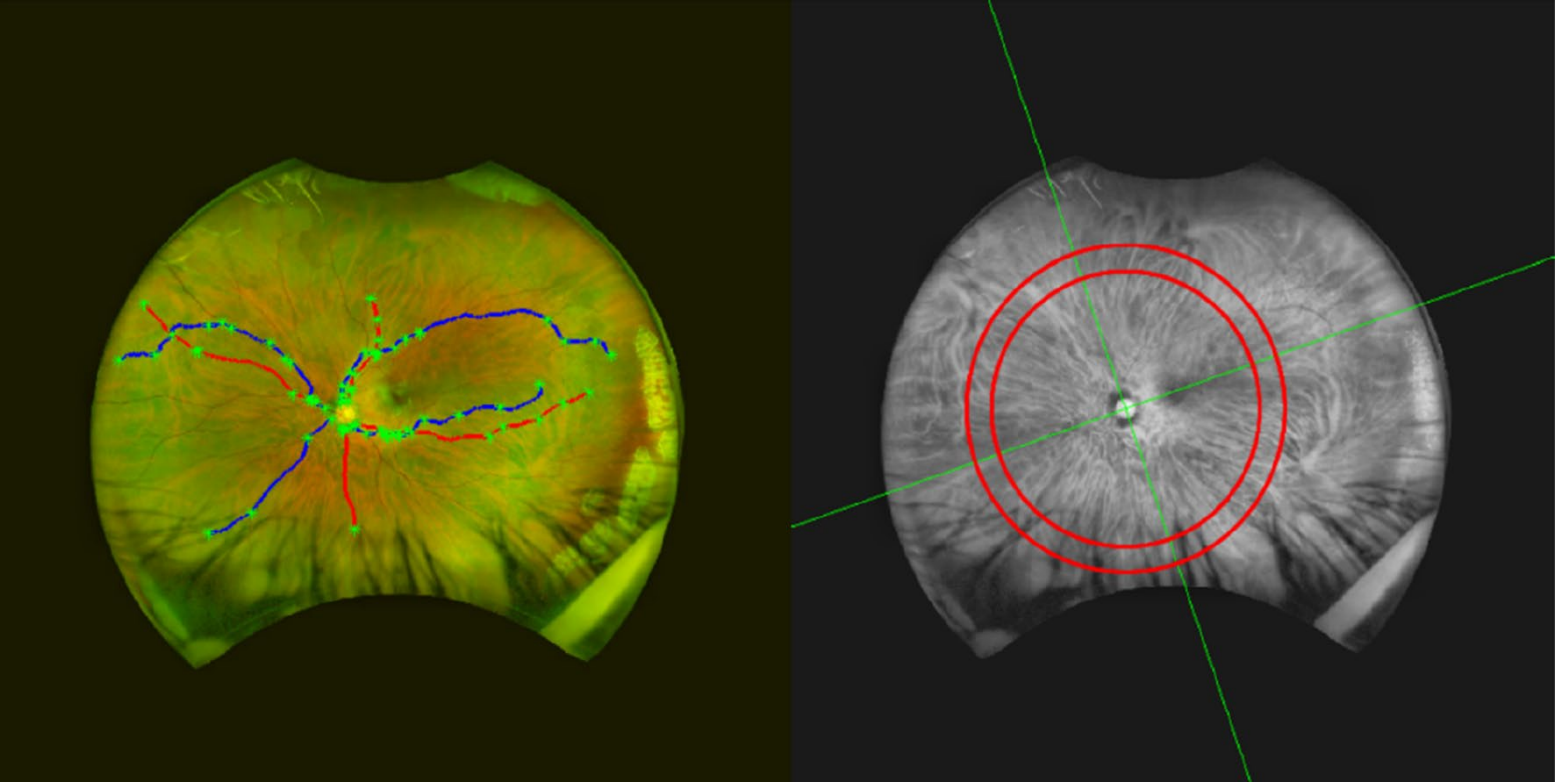
UWF imaging and analysis**.** Left: Example of the 8 vessels selected for analysis in a participant (2 from each quadrant). Veins are blue and arteries red, with the nodes (i.e., crossings, bifurcations, start and end points) plotted as green crosses. Right: The red channel of the same image separated into quadrants (green lines), with 6.5 to 8.5 optic disc radii marked with the red concentric circles.

Imaging of the fundus to examine the retinal vasculature was conducted using an Optos Daytona Ultra-Widefield Scanning Laser Ophthalmoscope (UWF-SLO) (Optos, Dunfermline, Scotland), which creates an image by scanning two laser light sources (635 nm and 532 nm)^25^. Two images of each eye were captured, and the best image was selected for analysis (determined by manual inspection of how clearly both the optic disc and fovea could be viewed and the amount of peripheral vasculature that was visible). UWF images were subjected to a stereographic projection that re-maps the image to account for the curvature at the back of the eye, conserving angular dimensions and enabling geometric measurements accurate to 2%^26^. The vasculature was then segmented using a semi-automatic technique^27–30^. Measures of vessel diameter were converted from pixels to millimetres using an algorithm (supplied by Optos and available under the DICOM standard) that accounts for a pixel’s global position on the retina with respect to the fovea^31,32^. For each image, a maximum of 8 vessels were selected for analysis—1 vein and 1 artery from each of the inferior nasal, superior nasal, inferior temporal, and superior temporal quadrants (Fig. 1). Quadrants were demarcated with a line from the centre of the optic disc to the centre of the fovea (both landmarks manually identified by author TP) and a second line perpendicular to this to separate the nasal/temporal hemispheres. The same vessels were selected each time for analysis in follow-up images, and all images were anonymised of participant identifiers to mask the investigator and not introduce selection bias. As vessels decrease in diameter as a function of their length (from the optic disc to the periphery), care was taken to create a standardised vessel width measure from within a specific location on the retina. Any pixel from a selected vessel which lay between 6.5 and 8.5 optic disc radii away from the optic disc centre was used for vessel width analysis.

Statistical analysis was performed using the MATLAB Statistics Toolbox (Release 2015a, The MathWorks, Inc., Natick, Massachusetts, United States). Normality of the data collected was tested using a selection of objective and subjective approaches. Where data was found to be normally distributed, a two-sample t-test was used to identify significant differences between the retinal parameters measured from each participant group. To determine the effects of potentially confounding variables in the retinal parameters measured, the data was analysed using multivariate linear regression models in correspondence with similar studies^17,18,33^. Despite a tendency in the literature to adjust for interocular variations within regression models, or to assume left and right eyes to have a high degree of inter-correlation, this study kept eye data separate to allow for lateral asymmetry, which has been found in RNFL thickness measures of healthy individuals^34^.

As per regression models put forward by comparable studies, *age* was considered as one of the possible predictors of RNFL thinning along with two binomial variables: *disease* (disease vs no disease) and *sex* (female vs male)^35,36^. However, to avoid overfitting of the RNFL thickness data, *age* and *sex* were included as confounders only when their inclusion in the multivariate modelling helped to better describe the data (i.e., had a larger R-squared value and was statistically significant). The results of this analysis can be found in Supplementary Tables S1, S2, S3 and S4. The *disease* coefficient produced by each model was then used to assess the effect of MS on the measured anatomical changes in the retina, along with its statistical significance.

To assess the robustness of each model, they were verified subjectively; the residuals from each model were tested for normality (to ensure a linear model is correct for characterising the data) via box plots, histograms, and normal probability plots. The models were also tested for robustness by running them with the most influential data points removed from the dataset; influential points were identified as those having a Cook’s distance which was greater than 3 times the mean Cook’s distance for the dataset from which it came^37^. Any anatomical changes seen at follow-up, in either imaging modality, were standardised as a rate-of-change per year- a technique mirroring that of another study which imaged participants longitudinally at irregular intervals^33^.

## Results

### Data collection

Baseline retinal scans were successfully attained for 72 participants with MS and 80 healthy volunteers. Length of disease (calculated from the initial date of diagnosis with MS) ranged from 1.7 to 38.5 years. Median age was slightly lower in the group of healthy volunteers (38 years) when compared to participants with MS (44 years). An equal number of male participants were scanned in both the healthy volunteer and MS groups (19 participants), although more female healthy volunteers were scanned than females with MS. Further information on participant demographics is displayed in Table 1.

**Table 1:** Participant demographics.

Characteristics	Participant group	
	Patients with MS (N = 72)	Healthy volunteers (N = 80)
Median age	44	38
Age range	20–79	23–73
Male	19	19
Female	53	61
Relapse-remitting	62	N/A
Primary progressive	2	N/A
Secondary progressive	7	N/A
Disease-modifying therapies*	46	N/A

OS, Left eye; OD, Right eye.

*These include: Fingolimod, Tecfidera, Dimethylfumerate, Copaxone, Plegridy, Alemtuzimab and Tysabri. One MS participant’s disease subtype was unable to be determined from their electronic correspondence.

Follow-up scans were attained for 69 participants with MS and 64 healthy controls. The number of imaging sessions for participants with MS ranged from 1 to 5, with the time between baseline and final scan ranging from 196 to 672 days. Healthy volunteers were scanned between 1 and 3 times, with the time between baseline and final imaging sessions ranging between 227 to 802 days. For a small number of participants, a good-quality OCT image capture was not successful: one healthy volunteer suffered from age-related macular degeneration and some individuals with MS were not able to sustain focus for the entire scanning procedure. Of the healthy volunteers included in this study, two fell pregnant during the time between baseline and follow-up scan. Both these participants presented with a significantly thicker RNFL when scanned during pregnancy: an anatomical change that has been reported, though scarcely, in the literature^38^. Due to this thickening of the RNFL, scans from during the pregnancy were excluded from follow-up analysis to maintain a consistent and fair approach to data handling. The final number of usable OCT datasets are shown in Supplementary Table S5. Supplementary Table S6 displays the number of UWF images for participants with MS and healthy volunteers that have visible blood vessels in each of the quadrants, which were then able to be segmented and used for analysis.

### Baseline OCT

Table 2 shows thinner RNFL thicknesses were measured for patients with MS in all regions, with statistical significance only lacking in the nasal inferior region. Reductions in macular volume were also statistically significant when compared to healthy volunteers. All attempts at modelling these data were found to be statistically significant (*p* < 0.01), except in the inferior nasal region (see Supplementary Table S1). *Disease* was found to be a statistically significant predictor (*p* < 0.05) of RNFL thinning in all regions except for inferior nasal (see Table 3). Although *age* was included when modelling RNFL data in all but one of the retinal regions, as well as in macular volume models, it was observed only to be a significant predictor of RNFL reductions globally (OS: − 0.18 μm/year, *p* value < 0.05; OD: − 0.19 μm/year, *p* value < 0.05), as well as in the superior nasal region of the left eye (− 0.38 μm/year, *p* < 0.05).

**Table 2:** Baseline OCT data.

Region	Mean RNFL thickness (µm ± SE)		The difference from mean HV baseline RNFL thickness in µm (95% CI)	
	Healthy volunteers		Participants with MS	
	OS	OD	OS	OD
Global	98 ± 1.2	99 ± 1.3	− 10 (− 14, − 5)***	− 11 (− 15, − 7)***
Temporal	70 ± 1.3	71 ± 1.5	− 11 (− 16, − 7)***	− 12 (− 17, − 7)***
Temporal inferior	145 ± 2.3	142 ± 2.4	− 15 (− 23, − 7)***	− 13 (− 22, − 5)**
Temporal superior	136 ± 1.9	136 ± 2.3	− 10 (− 17, − 3)**	− 9 (− 15, − 2)*
Nasal	74 ± 1.7	78 ± 2	− 8 (− 13, − 3)**	− 11 (− 16, − 6)***
Nasal inferior	110 ± 2.9	113 ± 3	− 5 (− 14, 4)	− 8 (− 17, 0)
Nasal superior	108 ± 2.3	99 ± 2.2	− 9 (− 17, − 1)*	− 9 (− 16, − 3)*

Mean macular volume (mm^3^ ± SE)		The difference from mean HV baseline macular volume in mm^3^ (95% CI)		
Healthy volunteers		Participants with MS		
OS	OD	OS	OD	
8.68 ± 0.04	8.66 ± 0.04	− 0.22 (− 0.35, − 0.08)**	− 0.3 (− 0.44, − 0.17)***	

The differences in mean baseline RNFL thickness and macular volume for MS participants when compared to healthy volunteers (HV), assessed using an unpaired samples Student’s t-test.

SE, Standard error; CI, Confidence interval; OS, Left eye; OD, Right eye.

*p < 0.05; **p < 0.01; ***p < 0.001.

**Table 3:** Results of linear regression modelling with baseline OCT data.

Variable	OS		OD	
	Coefficient of *disease*	*p* value	Coefficient of *disease*	*p* value
Global RNFL thickness (µm)	− 9	< 0.001	− 10	< 0.001
Temporal RNFL thickness (µm)	− 11	< 0.001	− 12	< 0.001
Temporal inferior RNFL thickness (µm)	− 14	< 0.001	− 12	< 0.01
Temporal superior RNFL thickness (µm)	− 10	< 0.01	− 8	< 0.05
Nasal RNFL thickness (µm)	− 7	< 0.01	− 10	< 0.001
Nasal inferior RNFL thickness (µm)	− 4	0.34	− 8	0.09
Nasal superior RNFL thickness (µm)	− 8	< 0.05	− 8	< 0.05
Macular volume (mm^3^)	− 0.207	< 0.01	− 0.29	< 0.001

The coefficient of *disease* and its associated *p* value is shown for each retinal region as well as for macular volume.

OS, Left eye; OD, Right eye.

### Baseline UWF-SLO

Table 4 displays vessel width measurements, which indicate that the participants with MS in this study had thinner retinal arteries and veins than healthy volunteers. Arterioles were measured as being significantly thinner in the nasal inferior quadrant of participants with MS. The same was only true of venules in right eyes. Mean vessel width graphs are provided in supplementary Figs. S1 to S4. All linear regression models for arterial width measurements were found to be statistically significant with exception of the superior nasal quadrant in both eyes and the superior temporal quadrant of the left eye (see Supplementary Table S2). The predictor *disease* correlated to a significantly reduced mean arterial width in the inferior nasal quadrant of MS participants when compared to the healthy volunteers (OS: − 0.0053 mm, *p* value < 0.05; OD: − 0.0079 mm, *p* value < 0.01), as seen in Table 5. When modelling venous width measurements, only data from the inferior nasal quadrant of the right eye produced a statistically significant model (see Supplementary Table S3). However, the predictor *disease* was again found to be statistically significant in  the inferior nasal quadrant, correlating to a reduced venous width in participants with MS (OS: − 0.0051, *p* value < 0.05; OD: − 0.0054, *p* value < 0.05).

**Table 4:** Baseline UWF-SLO data.

Region	Vessel type	Mean vessel width (mm ± SE)		The difference from mean HV baseline vessel width in mm (95% CI)	
		Healthy volunteers		Participants with MS	
		OS	OD	OS	OD
Temporal inferior	Artery	0.095 ± 0.002	0.096 ± 0.002	− 0.003 (− 0.009, 0.003)	− 0.005 (− 0.011, 0.001)
	Vein	0.114 ± 0.002	0.109 ± 0.002	− 0.004 (− 0.009, 0.001)	+ 0.001 (− 0.006, 0.007)
Temporal superior	Artery	0.104 ± 0.002	0.104 ± 0.002	− 0.003 (− 0.008, 0.003)	− 0.002 (− 0.007, 0.004)
	Vein	0.118 ± 0.003	0.118 ± 0.002	− 0.002 (− 0.009, 0.005)	0 (− 0.006, 0.005)
Nasal inferior	Artery	0.072 ± 0.002	0.070 ± 0.002	− 0.005 (− 0.01, − 0.001)*	− 0.007 (− 0.012, − 0.002)*
	Vein	0.084 ± 0.001	0.082 ± 0.002	− 0.005 (− 0.01, 0)	− 0.005 (− 0.01, 0)*
Nasal superior	Artery	0.079 ± 0.001	0.077 ± 0.001	− 0.001 (− 0.005, 0.003)	− 0.003 (− 0.007, 0.001)
	Vein	0.092 ± 0.001	0.092 ± 0.002	− 0.003 (− 0.007, 0.001)	− 0.004 (− 0.009, 0)

The difference in mean arterial and venous width for MS participants when compared to the baseline vessel width of healthy volunteers using an unpaired samples Student’s t-test, in four quadrants.

SE, Standard error; CI, Confidence interval; OS, Left eye; OD, Right eye.

*p < 0.05; **p < 0.01; ***p < 0.001.

**Table 5:** Results of linear regression modelling with baseline UWF-SLO data.

Region	Vessel type	OS		OD	
		Coefficient of *disease* (mm)	*p* value	Coefficient of *disease* (mm)	*p* value
Temporal inferior	Artery	− 0.0018	0.55	− 0.0044	0.15
	Vein	− 0.0042	0.12	1e−04	0.81
Temporal superior	Artery	− 0.0015	0.57	− 7e−04	0.8
	Vein	− 0.0021	0.56	4e−04	0.9
Nasal inferior	Artery	− 0.0053	< 0.05	− 0.0079	< 0.01
	Vein	− 0.0051	< 0.05	− 0.0054	< 0.05
Nasal superior	Artery	− 0.0005	0.78	− 0.0027	0.18
	Vein	− 0.0029	0.16	− 0.0043	0.08

The coefficient of *disease* and its associated *p* value is shown for each retinal quadrant.

OS, Left eye; OD, Right eye.

### Follow-up OCT

Table 6 displays the median rate of change in RNFL thickness found in each anatomical region. *Disease* was a statistically significant predictor in all models except the inferior temporal region in both eyes and the superior temporal region of the right eye (Table 7). The only region where *disease* was not shown to cause an increase in the annual rate of RNFL thinning was the inferior temporal region of the left eye. An annual global reduction of RNFL thickness in both eyes (OS: − 0.98, *p* < 0.001; OD: − 0.77, *p* < 0.01) was found. Macular volume was shown to reduce at a higher annual rate for individuals with MS, with *disease* being significant in both eyes (OS: − 0.047, *p* < 0.01; OD: − 0.042, *p* < 0.01).

**Table 6:** Median annual RNFL thickness and macular volume changes seen in the three participant groups.

Region	Median rate of change in RNFL thickness in µm/year (IQR)			
	Healthy volunteers		Participants with MS	
	OS	OD	OS	OD
Global	0 (1.31)	0.69 (0.98)	− 0.29 (2)***	0 (1.96)**
Temporal	0.7 (1.01)	0 (1.67)	0 (2.07)**	0 (1.74)
Temporal inferior	0 (1.7)	0 (1.84)	− 0.11 (3)	0 (2.76)
Temporal superior	0.31 (1.89)	0.65 (1.6)	0 (2.49)*	− 0.05 (3.67)
Nasal	0 (1.79)	0.76 (1.4)	− 0.63 (2.13)*	0 (2.36)
Nasal inferior	0.05 (2.31)	0.7 (1.8)	− 1.06 (2.4)***	0 (2.35)*
Nasal superior	0 (2.16)	0.41 (2.19)	− 0.53 (3)*	0 (2.46)

Median rate of change in macular volume in mm^3^/year (IQR)				
Healthy volunteers		Participants with MS		
OS	OD	OS	OD	
0.001 (0.092)	0.012 (0.086)	− 0.028 (0.077)**	− 0.02 (0.087)**	

OS, Left eye; OD, Right eye; ON, Optic neuritis; MS, Multiple sclerosis; SD, Standard deviation.

*p* values: * < 0.05; ** < 0.01; *** < 0.001.

**Table 7:** Coefficient values produced when modelling RNFL thickness and macular volume data produced from follow-up analysis.

Region	OS		OD	
	Coefficient of *disease* (µm/year)	*p* value	Coefficient of *disease* (µm/year)	*p* value
Global	− 0.98	< 0.001	− 0.77	< 0.01
Temporal	− 0.8	< 0.01	− 0.66	< 0.05
Temporal inferior	0.3	0.56	− 0.17	0.66
Temporal superior	− 1.01	< 0.05	− 1.02	0.08
Nasal	− 1.2	< 0.05	− 0.78	< 0.05
Nasal inferior	− 1.32	< 0.05	− 1.06	< 0.01
Nasal superior	− 1.11	< 0.05	− 1.13	< 0.01

	Macular volume data			
	Coefficient of *disease* (mm^3^/year)	*p* value	Coefficient of *disease* (mm^3^/year)	*p* value
Macular volume	− 0.047	< 0.01	− 0.042	< 0.01

OS, Left eye; OD, Right eye.

### Follow-up UWF-SLO

Over the time scale for which data was collected no models were deemed significant in any region for retinal blood vessel width.

## Discussion

The OCT results in this study resonate with previous reports in the literature^17,18^. Our linear regression modelling suggests that the differences seen between the groups were overwhelmingly due to *disease,* although in some models the predictors *age* and *sex* were also significant. Global differences (OS: − 9 µm, *p* < 0.001; OD: − 10 µm, *p* < 0.001) were consistent with a large multicentre study of 414 participants with MS, although larger than that reported by a recent systematic review and meta-analysis which saw a 7.41 µm (− 8·98 to − 5·83; *p* < 0·0001) thinner RNFL in 4109 eyes^16,39^. Direct comparisons to other studies are however limited by inter-operator and inter-instrument variability when measuring RNFL thickness^40^. This study’s results show a similar range of temporal RNFL thickness reduction (OS: − 11 µm, *p* < 0.001; OD: − 12 µm, *p* < 0.001) as presented by Antonio-Santos et al. (− 11 µm *p* < 0.0001) and Henderson et al. (− 12.8 µm to − 22.9 µm)^19,41^. Although low adjusted R-squared values produced when modelling baseline OCT data may indicate the absence of one or more confounders from the models, all models were found to be significant (*p* < 0.01), except when modelling inferior nasal region RNFL data, indicating that the models had descriptive value with *disease* being the most consistently significant predictor.

Longitudinal data provided less conclusive results in assessing the rate-of-change of RNFL thickness and macular volume. This is likely due to the smaller effect size; comparing results at baseline will show compounded differences which have occurred year-on-year, whereas annual changes are both smaller and more susceptible to small inaccuracies caused by erroneous segmentation. Changes that occur over these time periods may also be smaller than the resolution of the device used to capture images (i.e., 7 µm axial resolution for spectral domain OCT devices vs 1.1 to 2.9 µm reduction in global RNFL thickness every 2 years), resulting in only extreme changes being detected or considered clinically significant. The low rate-of-change values seen suggests that the pace with which anatomical changes occur in the retina is slow (approximately 6–7 years for a measurable change), regardless of disease status.

Differences were measured when comparing baseline vessel width from healthy volunteer and MS groups, although the degree of change was dependent on the region from which measurements were taken. Most notably, vessels in the nasal quadrants of the retina seem most affected in the MS group, being the only region that saw significant differences in vessel width. The location of the greatest vascular differences between these two groups is not the same as changes seen in the RNFL data. This may be because blood vessels in the temporal region form a more complex network with a greater number of bifurcations and vessel crossings as capillaries supply a large quantity of oxygenated blood to the macular. Such a complex network might lead to more errors in the segmentation of blood vessels and may therefore cover up any subtle changes in vessel width. Changes to vascularity in these areas of the retina may be better measured by OCT angiography (OCT-A), which has in recent years offered researchers the ability to non-invasively visualise the retinal microvasculature. Multiple studies have subsequently reported a reduced vascular density for patients with MS when compared to healthy controls in the peripapillary and/or macular regions of the retina^42–44^. OCT-A has also shown the potential to visualise pathological changes to the retinal anatomy earlier in the MS disease course than OCT alone^42,45^.

On the other hand, the differences between retinal quadrants may be an accurate representation of anatomical/pathological changes which occur within specific locations of the retina, or to the larger retinal blood vessels (rather than to the capillary supply as measured by OCT-A). Another study’s attempt to quantify retinal vascular changes by using peripapillary OCT scans found that vessels were significantly thinner in participants with MS^28^. However, no attempt was made to classify the vessels as arteries/veins or to separate the blood vessels into their respective anatomical quadrants. Although no other study has investigated the retinal vasculature in peripheral regions as our one has, the reduced vessel widths measured here seem complimentary to Bhaduri’s study of blood vessels in closer proximity to the optic disc^28^.

Longitudinal analysis showed no evidence of regression models being significant when attempting to describe the data and the resulting adjusted R-squared values were extremely small, with no single predictor being significant either. This may be due to a lack of power as changes in vessel width are small and there were low participant numbers, or the timescale was too short resulting in disease having no clear effect on the rate-of-change of vessel thinning. Alternatively, this modelling could suggest that changes to vessel anatomy occur early in the disease course along with initial inflammatory responses within the brain; the initial breach of the blood–brain barrier may be mirrored in the blood-retina barrier, and the acute inflammatory effects of this may cause a chronic reduction in blood vessel width.

Future studies should be designed to confirm the differences seen at baseline between healthy volunteer and MS vessel widths. The use of higher resolution imaging techniques (e.g., fundus photography and next generation UWF-SLO devices) may be able to discern changes in vessel width to a greater precision than those utilised in this study and thus confirm the reduction in vessel width seen in the other quadrants of the retina without unfeasibly high participant numbers. Higher precision devices may also be beneficial in identifying small changes that occur longitudinally throughout the disease course, and thus determine whether the vascular changes seen occur as a function of time. Incorporation of axial measurements should also be considered to improve accuracy of vessel width measures^31^.

To better understand the correlation of disease severity with anatomical changes in the retina, clinical information including EDSS/Multiple Sclerosis Functional Composite (MSFC) and MRI analysis would be an asset in stratifying participants. A study that used said measures found a reduced rate of axonal thinning is associated with “no evidence of disease activity” in patients with MS, indicating that the rate of thinning is dependent on the disease status of an individual^46^. Finally, as segmentation of the deeper layers of the retina becomes more widely available with better lateral image resolution and more reliable segmentation algorithms, analysis of retinal layers other than the RNFL (e.g., Ganglionic layer) as well as the highly vascular Choroid layer could offer greater insight into changes to the retina associated with disease. Furthermore, statistical analysis which incorporates both RNFL and vessel data to explore correlations between the two could help researchers develop a more comprehensive understanding of how the retina changes with respect to disease severity.

One limitation of this study is the small timescale over which scans were taken relative to the potential length of disease course, especially given the slow rate at which changes to the retinal anatomy appear to occur^5^. Prospective investigations must look to determine whether changes occur over the entire disease course or are only the result of initial disease mechanisms. Linear regression models may however be ineffective at describing longitudinal changes over larger timescales, as thinning may plateau over time. As such, more sophisticated statistical analysis is likely needed to characterise the data; such analysis should also correct for multiple testing (due to the retina being analysed in four different regions) and investigate inter-ocular correlations so as not to increase the risk of false positives. Our study, though exploratory, is limited in this aspect.

Another potential limitation of our study is the mixture of MS subtypes in the patient cohort. While Relapse Remitting made up the majority (n = 62) there were a small number of Primary Progressive (n = 2) and Secondary Progressive patients (n = 7), which could have affected our investigatory analysis into changes occurring in the retina in MS. A more targeted approach to participant recruitment would aid future investigations into the potential differences in changes to the retinal anatomy between subtypes of MS.

Evidence suggests that retinal vessel topology is influenced by diseases other than MS e.g., Diabetes Mellitus, Cardiovascular Disease and Alzheimer’s Disease^11^. We did not analyse this in our exploratory study and it is another potential limitation. Though MS data was compared to Healthy Volunteers, any future studies should either expand the exclusion criteria to include individuals with such diseases, or such diseases should be controlled for in the statistical analysis through taking a thorough medical history and exploring patient records. Equally, individuals with current or recent relapses and patients being treated with steroids at the time of investigation should be considered for exclusion and as such, may limit this study.

## Conclusion

This study not only reproduced results consistent with previous reports on the reduction of RNFL thickness and macular volume in patients with MS, but provides the first evidence of changes to the retina’s peripheral vasculature caused by MS. This discovery, alongside recent evidence from OCT-A, suggests there is a significant vascular component to the retinal changes seen in MS. Future studies should further investigate changes in retinal blood vessels to determine when in the MS disease course these changes occur and whether they have prognostic or diagnostic potential.

## Supplementary Information

Supplementary Information.

## Data Availability

The dataset generated and analysed during this study is available from author T.M on reasonable request.
