# Supplementary material for: Multi-modal retinal scanning to measure retinal thickness and peripheral blood vessels in multiple sclerosis
Source: Sci Rep. 2022 Nov 28;12:20472. doi: 10.1038/s41598-022-24312-4 (PMC9705292; doi:10.1038/s41598-022-24312-4)
Supplement: Supplementary file 1 — Supplementary Information. [file 41598_2022_24312_MOESM1_ESM.pdf]

Supplementary Information:

Multi-modal retinal scanning to measure retinal thickness and peripheral blood vessels in Multiple Sclerosis

Thomas Pearson[1*], Yingdi Chen[1,2], Baljean Dhillon[1,3], Siddharthan Chandran[1,2], Jano van Hemert[4], Tom MacGillivray[1]

[1]Centre for Clinical Brain Sciences, University of Edinburgh, Edinburgh, UK

[2]Anne Rowling Regenerative Neurology Clinic, University of Edinburgh, Edinburgh, UK

[3]Princess Alexandra Eye Pavilion, NHS Lothian, Edinburgh, UK

[4]Optos plc, Dunfermline, KY11 8GR, UK

*Corresponding author: 03pearsont@gmail.com

Analysis of Multivariate Modelling

For baseline RNFL thickness data, *disease* and *age* produced the multivariate model which was most representative of global thickness in both eyes (Table S1). This was also the case in all nasal regions. However, the temporal regions were less consistent e.g., the temporal region itself was best described by the univariate model of *disease* as the only predictor. The only region for which *sex* added predictive power was the inferior temporal of the left eye (adjusted R-squared=0.09, p<0.01), but macular volume was best described by all three predictors in both eyes.

	**Variables included in model**					
	Disease, age, sex		*Disease, age*		*Disease*	
**Retinal region**	**OS**	**OD**	**OS**	**OD**	**OS**	**OD**
Global	0.144***	0.187***	0.148***	0.193***	0.128***	0.166***
Temporal	0.14***	0.15***	0.142***	0.153***	0.146***	0.154***
Temporal Inferior	0.092**	0.075**	0.089***	0.078**	0.089***	0.064**
Temporal Superior	0.043*	0.055*	0.049*	0.06**	0.052**	0.042*
Nasal	0.08**	0.111***	0.084**	0.118***	0.067**	0.105***
Nasal Inferior	0.003	0.014	0.011	0.022	0.003	0.02
Nasal Superior	0.066**	0.062*	0.068**	0.063**	0.034*	0.052**
Macular Volume	0.075**	0.146***	0.068**	0.137***	0.066**	0.13***

p-values: * <0.05, **<0.01, ***<0.001

Table S1: Results from linear regression modelling of OCT data**.** Adjusted R-squared values for three linear regression models using the predictors *age*, *sex,* and *disease*. The model highlighted in grey for each region was selected for coefficient analysis. OS = left eye, OD = right eye.

The adjusted R-squared values when modelling for baseline arterial and venous width in each peripapillary quadrant of the retina are shown in Tables S2 and S3 respectively. Much like the regression models created for OCT data, adjusted R-squared values were generally very small and the models which best describe vessel width varied from region to region and between the left and right eye. Despite this, arterial width models were found to be significant with exception of the superior nasal quadrant in both sets of eyes and the superior temporal quadrant

of the left eye. In the venous width modelling, only the univariate model for inferior nasal data in the right eye was found to have significance.

	**Variables included in model**					
	Disease, age, sex		*Disease, age*		*Disease*	
**Retinal region**	**OS**	**OD**	**OS**	**OD**	**OS**	**OD**
Temporal Inferior	0.041*	0.038	0.045*	0.042*	0	0.017
Temporal Superior	0.019	0.049*	0.027	0.042*	-0.001	-0.005
Nasal Inferior	0.03	0.095**	0.039	0.086**	0.046*	0.061**
Nasal Superior	-0.002	-0.005	-0.006	0	-0.007	0.006

p-values: * <0.05, **<0.01, ***<0.001

Table S2: Results from linear regression modelling of UWF-SLO arterial data. Adjusted R-squared values for three linear regression models. The model highlighted in grey for each quadrant was selected for coefficient analysis. OS = left eye, OD = right eye.

	**Variables included in model**					
	Disease, age, sex		*Disease, age*		*Disease*	
**Retinal region**	**OS**	**OD**	**OS**	**OD**	**OS**	**OD**
Temporal Inferior	0.032	-0.019	0.004	-0.01	0.012	-0.008
Temporal Superior	-0.004	0.014	-0.01	0.002	-0.004	-0.008
Nasal Inferior	0.042	0.027	0.033	0.035	0.028	0.027*
Nasal Superior	-0.0066	0.0016	0.001	0.008	0.008	0.016

p-values: * <0.05, **<0.01, ***<0.001

Table S3: Results from linear regression modelling of UWF-SLO venous data. Adjusted R-squared values for three linear regression models. The model highlighted in grey for each quadrant was selected for coefficient analysis. OS = left eye, OD = right eye

Retinal region	Variables included in model					
	Disease, age, sex		*Disease, age*		*Disease*	
	**OS**	**OD**	**OS**	**OD**	**OS**	**OD**
Global	0.07 **	0.064 *	0.078 **	0.07 **	0.084 ***	0.045 *
Temporal	0.048 *	0.022	0.056 *	0.025	0.064 **	0.033 *
Temporal Inferior	-0.017	-0.011	-0.011	-0.013	-0.006	-0.007
Temporal Superior	0.014	0.008	0.023	0.014	0.028 *	0.018
Nasal	0.034	0.022	0.033	0.028	0.036 *	0.023
Nasal Inferior	0.053 *	0.069**	0.046 *	0.077 **	0.03 *	0.034 *
Nasal Superior	0.012	0.052 *	0.018	0.053 *	0.026 *	0.044 *
Macular Volume	0.066 *	0.083 **	0.065 **	-0.073 **	0.071 **	0.08 **

p-values: * <0.05, **<0.01, ***<0.001

Table S4: Adjusted R-squared values when modelling annual changes in RNFL thickness and macular volume. The model highlighted in grey for each region was selected for coefficient analysis. OS = left eye, OD = right eye, ON = optic neuritis, MS = multiple sclerosis, IQR = interquartile range

The adjusted R-squared values for regression models of longitudinal changes in RNFL thickness and macular volume are shown in Table S4. As was true with baseline regression models, despite low adjusted R-squared values there were significant models produced for multiple regions: in 4 regions there lacks any of evidence effect, three of which are from the temporal regions where data was best represented by a univariate model with *disease* acting as the lone predictor. However, generally left eye data was better represented by univariate models with only the inferior nasal region including the predictors *age* and *sex*, whereas right eye data was better described by multivariate models in 5 of the 8 datasets.

Both arterial and venous data was evaluated with linear regression models to eliminate the possible effects of confounding predictors in the median rate-of-change analysis, but over the time scale for which data was collected no models were deemed statistically significant in any region.

Participant Demographics

	Participant group			
	Participants with MS		Healthy volunteers	
	OS	OD	OS	OD
Number of scans	60	59	72	71

Table S5. Number of OCT baseline images for each participant group which were of good enough quality for analysis. Participants with MS and a clinical history of optic neuritis are not included. OS = left eye, OD = right eye

		Participant group			
		Healthy Volunteers		Participants with MS	
Retinal Region	Vessel type	OS	OD	OS	OD
Temporal Inferior	Artery	64	57	54	53
	Vein	65	62	54	55
Temporal Superior	Artery	68	70	56	61
	Vein	66	70	58	61
Nasal Inferior	Artery	55	58	35	46
	Vein	57	64	37	50
Nasal Superior	Artery	68	70	54	58
	Vein	69	72	55	59

Table S6: Number of UWF-SLO scans. The number of images for each group from which arterial and venous data could be taken, from each of the four retinal quadrants.

Vessel width data

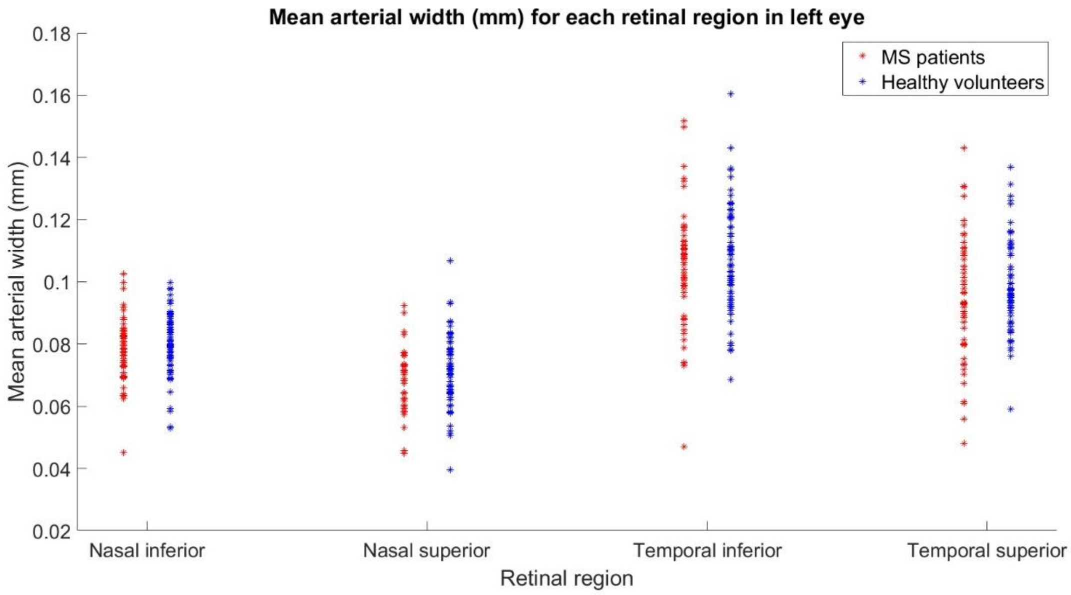

Figure S1: Mean vessel width (mm) for arteries in each of the four retinal quadrants in the left eye. Red represents MS patients. Blue represents healthy volunteers

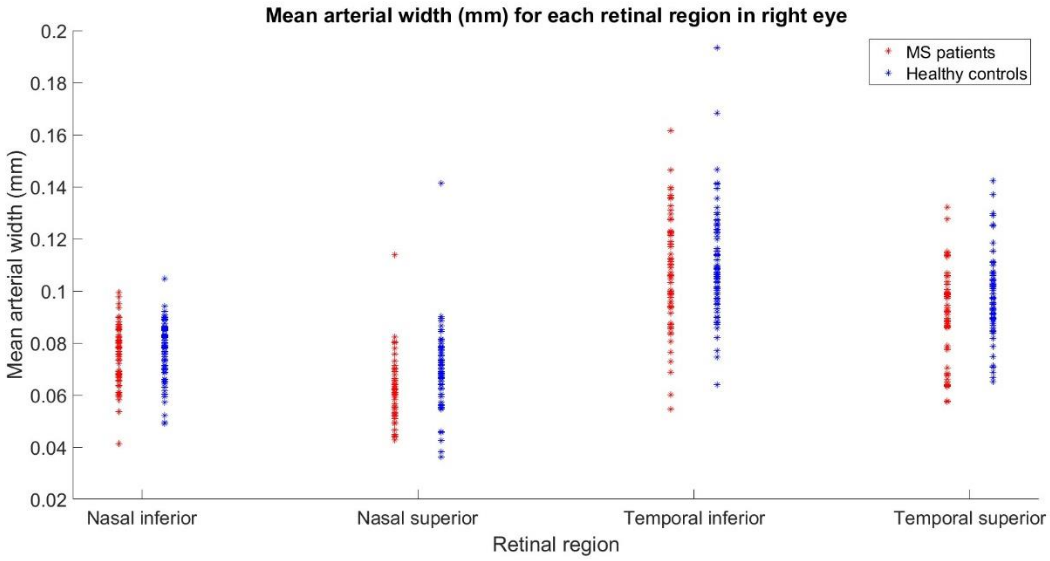

Figure S2: Mean vessel width (mm) for arteries in each of the four retinal quadrants in the right eye. Red represents MS patients. Blue represents healthy volunteers

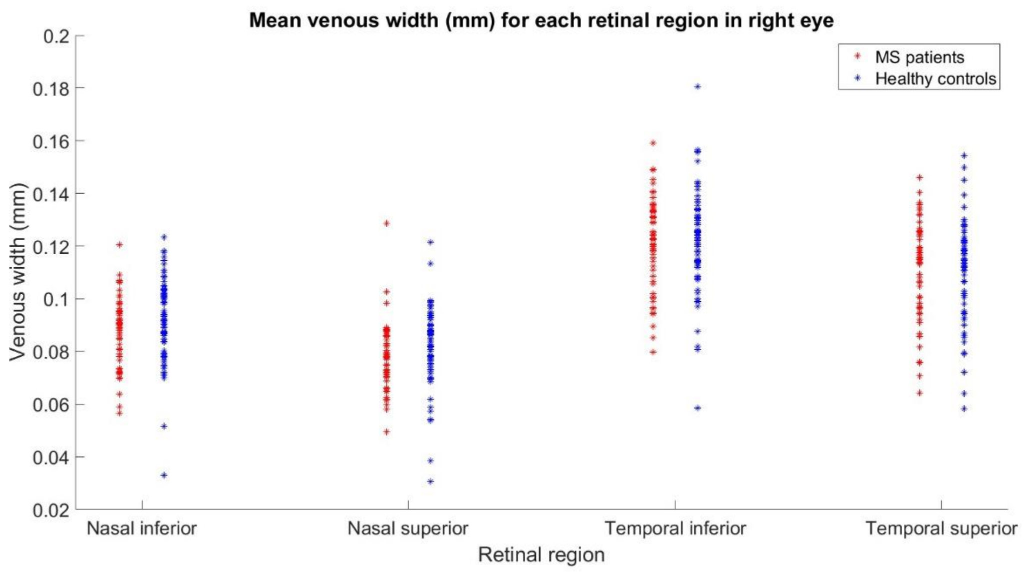

Figure S3: Mean vessel width (mm) for veins in each of the four retinal quadrants in the right eye. Red represents MS patients. Blue represents healthy volunteers

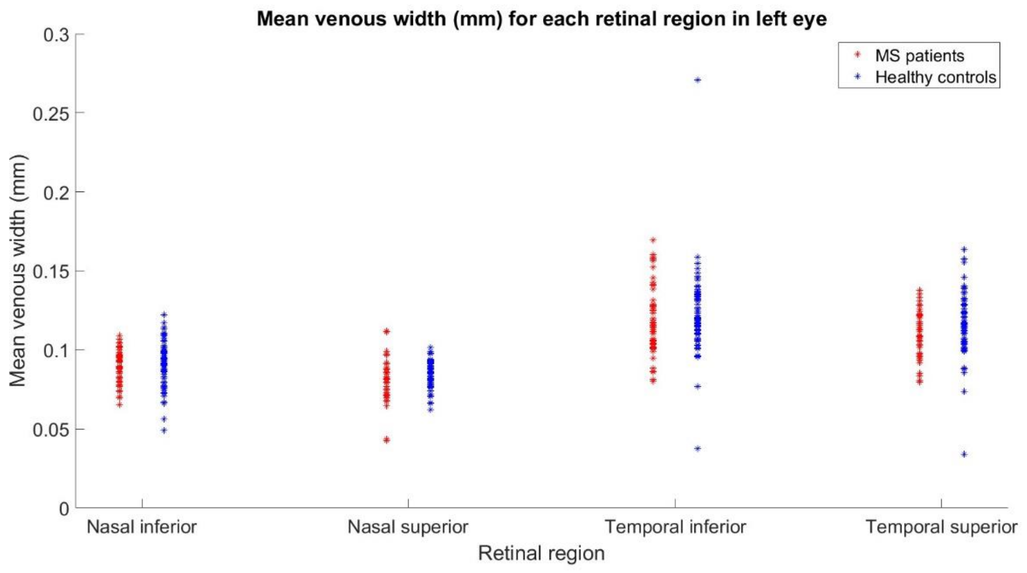

Figure S4: Mean vessel width (mm) for veins in each of the four retinal quadrants in the left eye. Red represents MS patients. Blue represents healthy volunteers
